# Retrograde Cricopharyngeus Dysfunction effectively treated with low dose botulinum toxin. A case report from Italy

**DOI:** 10.3389/fneur.2023.1238304

**Published:** 2023-08-09

**Authors:** Luca Pavesi, Cecilia Balzano, Simone Mauramati, Carla Giudice, Mauro Fresia, Massimiliano Todisco, Enrico Alfonsi, Giuseppe Cosentino

**Affiliations:** ^1^Independent Researcher in Pharmaceutical Chemistry and Technology and in Nutritional Sciences, Novara, Italy; ^2^Department of Otorhinolaryngology, Fondazione IRCCS Policlinico San Matteo, Pavia, Italy; ^3^Department of Brain and Behavioral Sciences, University of Pavia, Pavia, Italy; ^4^IRCCS Mondino Foundation, Pavia, Italy

**Keywords:** Retrograde Cricopharyngeus Dysfunction (R-CPD), R-CPD, inability to burp, botulinum injection, abelchia, onabotulinum toxin-A, cricopharyngeus muscle, case report

## Abstract

A large constellation of hitherto unexplained symptoms including inability to burp, gurgling noises from the chest and lower neck, abdominal bloating, flatulence, painful hiccups and emetophobia was defined as Retrograde Cricopharyngeus Dysfunction (R-CPD) in 2019. First choice treatment of R-CPD involves injection of botulinum toxin into the cricopharyngeus muscle under local or general anesthesia. This treatment has been found to be effective in the vast majority of subjects, with limited adverse events and prolonged therapeutic effects. Notwithstanding, R-CPD is still a poorly understood and underestimated disease, and a specific therapeutic dosage range of botulinum toxin (BT) has not been yet established. In this report, we describe the first case of R-CPD diagnosed in Italy, successfully treated with unilateral, anesthesia-free injection of 10 units of onabotulinum toxin into the cricopharyngeus muscle, representing the lowest dose reported to date.

## 1. Introduction

The inability to burp has been described for the first time in 1987 by Kahrilas et al. (1). Since then, only sporadic case reports (2–4) have been reported until the publication of a case series of 51 patients by Bastian et al. (5), who first defined the Retrograde Cricopharyngeus Dysfunction (R-CPD) syndrome. This disease is characterized by abelchia, socially awkward gurgling noises from the chest and lower neck, excessive abdominal bloating, flatulence, painful hiccups and, often, emetophobia (5). R-CPD is a very little-known disorder, and it is often misdiagnosed as a gastrointestinal disease, such as irritable bowel syndrome (IBS). Sometimes the patients themselves, driven by the persistence and severity of the symptoms, may get to guess the diagnosis while looking for information on the internet or obtaining clues from online patients’ communities. Based on the available literature, the treatment of R-CPD consists in the injection of 30 –100 units of onabotulinum toxin into the cricopharyngeus muscle (that represents the main portion of the Upper Esophageal Sphincter, UES) under local or general anesthesia (5–12). According to medium-term data published so far (6–8, 11, 12), the BT is effective in most subjects, often with prolonged therapeutic effects, lasting much longer than expected when treating other muscle districts. Here we describe the first case of R-CPD diagnosed in Italy, successfully treated with unilateral, anesthesia-free injection of 10 units of onabotulinum toxin-A into the cricopharyngeus muscle, representing the lowest dose reported to date.

## 2. Case description

A 28 year-old female patient suffered from both abdominal swelling and pain, gurgling noises, painful hiccups, nausea and flatulence since she was a child. She had been also experiencing chest pain and difficulty breathing from the age of 18. She was diagnosed with celiac disease when she was 7 year-old, but despite adopting a gluten-free diet her symptoms never improved. She was examined by several gastroenterologists who attributed her symptoms to celiac disease or other gastrointestinal disorders (such as IBS, dysbiosis, *Helicobacter pylori* infection), as well to psychiatric disorders. Prescriptions of various drugs, including antacids, simethicone, rifampicin, levosulpiride, charcoal, neuroleptics and benzodiazepines were not successful at all. Several previous esophagogastroduodenoscopies had shown no abnormalities, while abdominal ultrasound scans were often impracticable due to intestinal bloating. Suspicion of dolichocolon was ruled out by an abdominal CT scan. The patient herself, foreseeing that the cause of the symptoms could be her congenital inability to belch, came across Bastian’s publications, learning about R-CPD, whose diagnostic criteria perfectly matched her symptoms. The patient successfully contacted some European physicians willing to treat her, until she found the willingness of the IRCCS Mondino Foundation of Pavia, Italy (hereinafter Mondino Foundation) to take care of her.

## 3. Diagnostic assessment and treatment

In mid-March 2023, the patient underwent a single-day multidisciplinary assessment at the Mondino Foundation, that consisted in a speech therapy assessment, ENT evaluation with Fiberoptic Endoscopic Evaluation of Swallowing (FEES), neurological and neurophysiological evaluation.

**Figure 1:**
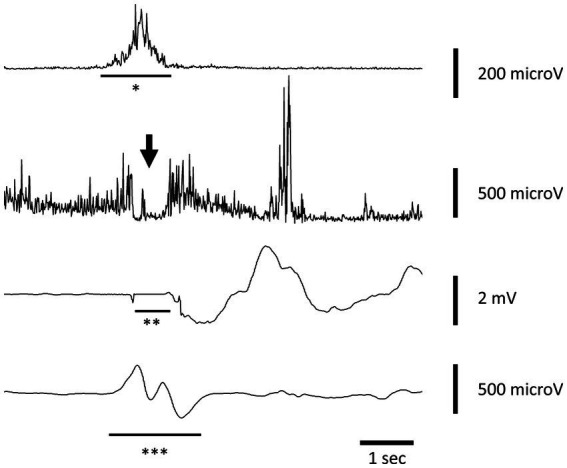
Electrokinesiographic/electromyographic study of swallowing in our patient. A representative trace recorded during voluntary swallowing of 12 cc of water. The first channel records the submental-suprahyoid electromyographic activity (*SHEMG) during both the oral and pharyngeal phase of swallowing using surface electrodes. The second channel records the electromyographic pause of the cricopharyngeal muscle (CPEMG) in the left portion during the pharyngeal (indicated by the arrow) phase of swallowing with a percutaneous monopolar needle (the same used to inject botulinum toxin). The third channel records breathing activity by means of a nasal cannula connected to a piezoelectric transducer. Negative and positive defections represent inspiration and expiration, respectively. An isoelectric line preceded and followed by two small notches indicates the swallowing-related apneic pause (**AP) due to the closure of the nasal choanae by the soft palate. The fourth channel records the laryngeal-pharyngeal mechanogram (***LPM) during the pharyngeal phase of swallowing using a piezoelectric transducer applied to the skin over the cricothyroid membrane.

At the speech therapy evaluation, the morpho-functional examination of the oropharyngeal structures was normal. Swallowing tests of liquids (water) and semi-solid foods were also carried out, finding in both cases good laryngeal excursion and absence of indirect signs of laryngeal penetration/aspiration. ENT and neurological examinations showed no abnormalities. FEES was performed with evidence of symmetrical hypertrophy of the base of the tongue and slight hyperaemia of the interarytenoid region. The patient underwent also an electrokinesiographic examination of the oral and pharyngeal phases of swallowing of water (3 and 12 cc) and jelly (3 and 12 cc) boluses. The electrokinesiographic parameters examined were normal (Figure 1) also including the cricopharyngeus muscle electromyographic pause during the pharyngeal phase of swallowing (13).

Since any other possible cause of the symptoms was ruled out, R-CPD was diagnosed based on patient’s anamnesis, clinical symptoms, and diagnostic results, representing to our knowledge the first reported case in Italy.

**Figure 2:**
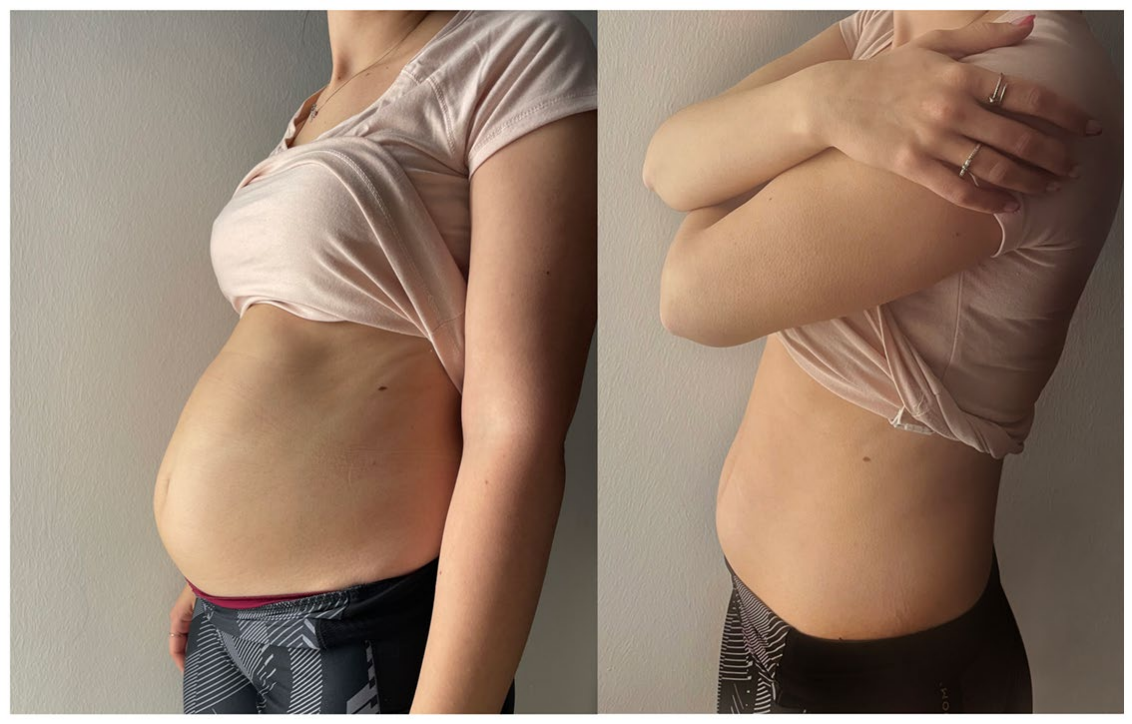
Photographs showing the dramatic improvement in abdominal swelling observed in the patient. The left photo was taken before BT administration, the right one was taken 11 days after BT administration.

On the same day of the evaluations, the patient underwent off-label injection of onabotulinum toxin-A into the cricopharyngeal muscle after informed consent was obtained. A low dosage of 10 units of onabotulinum toxin-A diluted in 0.1 mL of physiological solution was percutaneously injected under electromyographic guidance in the left posterior lateral portion of the CP muscle in one single spot. After ice spray was locally applied (without local anesthesia), the cannula needle (Ambu® Neuroline Inoject 38 mm/1.5″ length x 0.38 mm/26 Gauge Calibre, Ambu A/S, Denmark) was inserted 1 cm lateral to the palpable border of the cricoid cartilage with an inclination of approximately 45 degrees, to a depth of approximately 2 cm. The correct position of the needle was confirmed by the presence of sustained tonic muscle activity and of the electromyographic pause lasting a few hundred ms which was observed during swallowing and followed by a transient increase in the muscle tonic activity (muscle squeezing). To ensure not inoculating the posterior cricoarytenoid (PCA) muscle the patient was asked to make a sniff, that would result in a burst of EMG signal indicating incorrect location of the needle. The whole procedure lasted about 5 min and was well tolerated by the patient. Already the next evening after the administration, the patient emitted the first weak belching, followed in the next days, by more and more consistent burps together with a significant reduction in abdominal swelling and gurgling, as well as in all the other symptoms. Figure 2 shows the dramatic improvement in abdominal swelling observed 11 days after administration.

At the time of this writing (4 months after treatment), the symptoms have not reappeared, and no significant adverse events were reported, except for a slight and occasional dysphagia with some solid foods that lasted about 2 weeks after treatment. An overall judgment about global QOL was assessed by asking the patient to indicate a number on a scale ranging from 100 to 0, where 100 is labeled ‘Perfect quality of life,’ and 0 is labeled ‘Might as well be dead’ (14). The patient reported a score of 30 concerning the period immediately preceding the treatment, and a score of 100 at 1, 2, 3, 4 months follow up. Written informed consent was obtained from the patient for the publication of potentially identifying images and clinical details.

## 4. Discussion

Four case reports of inability to burp were published (1–4) prior to the identification of the R-CPD syndrome made by Bastian in 2019 (5). None of these patients was treated with botulinum toxin injection, which had hitherto only been used for antegrade dysfunction of the cricopharyngeus muscle (15–17). Bastian first described the efficacy of onabotulinum toxin injection into the cricopharyngeus muscle in 51 R-CPD patients using a dose of 50 units. All the treated patients were able to belch 24 to 48 h after injection.

Most studies published so far report a total dose range between 50 and 100 units of onabotulinum toxin injected into 1 or 2 different locations on both sides. To identify the most effective dose of onabotulinum toxin, Karagama (8) retrospectively analyzed clinical outcomes of 72 patients treated with three different dosages, 50, 75 or 100 units, coming to the conclusion that the higher dose could lead to a faster response, within 48 h from injection, and result in a longer treatment duration.

Only very recently, a retrospective study by Doruk and Pitman (12) showed that injection of a lower 30 units dose of BT, unilaterally injected into the cricopharyngeus muscle in 3 different spots, could be highly effective and safe.

According to recent literature on this topic, the symptoms completely disappear in most patients after the first treatment, although the effect of the botulinum toxin can wear off after 3 months and in many cases an additional injection is needed only after a longer period (5, 6, 11). It has been hypothesized that BT would act by relaxing the UES thus allowing the spontaneous expulsion of gas and the restoration of those mechanisms underlying belching. Even when the action of BT begins to wear off over time, the re-established neurophysiological mechanisms underlying belching could still work preventing the recurrence of symptoms (8).

Routinely, BT is performed via esophagoscopy under local or general anesthesia in an outpatient setting (5, 6, 8, 9). Wajsberg (7) proposed an electromyography-guided percutaneous approach involving the bilateral injection of BT into the cricopharyngeus muscle for the treatment of R-CPD. This method proven to be very accurate, can be easily performed in an outpatient setting, and limits possible complications due to general anesthesia. Doruk and Pitman (12) used a transcervical lateral approach for unilateral BT injection in an outpatient setting, showing a lower success rate than operating room injection but a better safety and tolerability profile. Recently, Xie (10) described an innovative method for the injection of botulinum toxin into the cricopharyngeus muscle under ultrasound, catheter balloon, and electromyographic guidance.

To our knowledge, here we describe the first patient diagnosed with R-CPD in Italy, and her positive clinical outcome after treatment with a very low dose of onabotulinum toxin (10 units/0.1 mL) injected unilaterally into the CP muscle in one single spot. The choice to use a low BT dose aimed at minimizing the possible onset of adverse events (e.g., transient dysphagia, acid brash upon belching, shortness of breath with exertion), taking into account the possibility of increasing the dose in subsequent treatment sessions. Since we commonly use low doses of botulinum toxin injected into one side to treat antegrade dysfunction of the CP muscle in patients with neurogenic dysphagia (18), we decided to follow the same protocol for the retrograde dysfunction. Following the administration of this low BT dose, the patient completely resolved the symptoms of R-CPD within a few weeks, without developing significant adverse events, except for a transient and mild dysphagia for solid foods. Noteworthy, the injection was performed on an outpatient basis without anesthesia, preventing its possible related complications. Some considerations and limitations of the study must be taken into account. First, a placebo effect cannot be completely excluded, though this seems very unlikely considering the long clinical history, the many treatments tried in the past and the long duration of the beneficial effects in our patient. Second, pharyngoesophageal manometry with belch provocation, that represents a useful diagnostic tool for R-CPD (9), was not performed in our patient. Further studies with longer clinical follow-ups are needed to confirm the efficacy of a low-dose, unilateral, EMG-guided BT injection into the CP muscle for treatment of R-CPD. Longer follow-up studies assessing the duration of treatment over time are also needed to clarify whether different physiological mechanisms could be involved in different patients subgroups to explain the response to treatment, including direct muscle relaxation effect and/or re-establishment of the physiological belching reflex. Finally, future studies should be carried out to assess whether different treatment approaches of R-CPD may lead to different outcomes for different patients, both in terms of efficacy, duration and safety of treatment.

## 5. Patient perspective

The patient referred that her quality of life significantly improved after BT injection. She reported that the gurgling disappeared 2 days after the injection and the rest of the symptoms are still absent 4 months after administration.

## Data availability statement

The original contributions presented in the study are included in the article/supplementary material, further inquiries can be directed to the corresponding author.

## Ethics statement

Ethical review and approval was not required for the study on human participants in accordance with the local legislation. The patients/participants provided their written informed consent to participate in this study. Written informed consent was obtained from the individual(s) for the publication of any potentially identifiable images or data included in this article.

## Author contributions

LP and CB: first drafting of the manuscript. GC: critical revision of the manuscript and patient treatment. GC, SM, CG, and MF: technical diagnostic assessment. GC, EA, SM, and CG: patient diagnosis. All authors contributed to manuscript revision, read, and approved the submitted version.

## Conflict of interest

The authors declare that the research were conducted in the absence of any commercial or financial relationships that could be construed as a potential conflict of interest.

## Publisher’s note

All claims expressed in this article are solely those of the authors and do not necessarily represent those of their affiliated organizations, or those of the publisher, the editors and the reviewers. Any product that may be evaluated in this article, or claim that may be made by its manufacturer, is not guaranteed or endorsed by the publisher.

## References

[1] Kahrilas PJ Dodds WJ Hogan WJ. Dysfunction of the belch reflex. A cause of incapacitating chest pain. Gastroenterology. (1987) 93:818–22. doi: 10.1016/0016-5085(87)90445-8 3623025

[2] Waterman DC Castell DO. Chest pain and inability to belch. Gastroenterology. (1989) 96:274–5. doi: 10.1016/0016-5085(89)90822-6 2909435

[3] Tomizawa M Kusano M Aoki T Ohashi S Kawamura O Sekiguchi T. A case of inability to belch. J Gastroenterol Hepatol. (2001) 16:349–51. doi: 10.1046/j.1440-1746.2001.02333.x, PMID: 11339431

[4] Sato H Ikarashi S Terai S. A rare case involving the inability to belch. Intern Med. (2019) 58:929–31. doi: 10.2169/internalmedicine.1908-18, PMID: 30449811 PMC6478983

[5] Bastian RW Smithson ML. Inability to belch and associated symptoms due to retrograde cricopharyngeus dysfunction: diagnosis and treatment. OTO Open. (2019) 3:1–7. doi: 10.1177/2473974X19834553, PMID: 31236539 PMC6572913

[6] Hoesli RC Wingo ML Bastian RW. The long-term efficacy of botulinum toxin injection to treat retrograde cricopharyngeus dysfunction. OTO Open. (2020) 4:1–6. doi: 10.1177/2473974X20938342, PMID: 32647778 PMC7325547

[7] Wajsberg B Hoesli RC Wingo ML Bastian RW. Efficacy and safety of electromyography-guided injection of botulinum toxin to treat retrograde cricopharyngeus dysfunction. OTO Open. (2021) 5:1–6. doi: 10.1177/2473974X21989587, PMID: 33598599 PMC7863157

[8] Karagama Y. Abelchia: inability to belch/burp-a new disorder? retrograde cricopharyngeal dysfunction (RCPD). Eur Arch Otorhinolaryngol. (2021) 278:5087–91. doi: 10.1007/s00405-021-06790-w, PMID: 33893849 PMC8553696

[9] Oude NRAB Snelleman JA Oors JM Kessing BF Heuveling DA Schuitenmaker JM. The inability to belch syndrome: a study using concurrent high-resolution manometry and impedance monitoring. Neurogastroenterol Motil. (2022) 34:e14250. doi: 10.1111/nmo.14250, PMID: 34435723 PMC9285907

[10] Xie M Wen H Dou Z. Case report: a case of novel treatment for retrograde cricopharyngeal dysfunction. Front Neurol. (2022) 13:1005655. doi: 10.3389/fneur.2022.1005655, PMID: 36619911 PMC9811257

[11] Silver JA Roy CF Young J Kost KM. Retrograde cricopharyngeus dysfunction: a canadian experience fueled by social media. Ear Nose Throat J. (2023):1455613231162203. doi: 10.1177/01455613231162203, PMID: 36864723

[12] Doruk C Pitman MJ. Lateral transcervical in-office botulinum toxin injection for retrograde cricopharyngeal dysfunction. Laryngoscope. (2023):871. doi: 10.1002/lary.30871, PMID: 37421251

[13] Alfonsi E Todisco M Fresia M Tassorelli C Cosentino G. Electrokinesiographic study of oropharyngeal swallowing in neurogenic dysphagia. Dysphagia. (2023) 38:543–57. doi: 10.1007/s00455-021-10336-x, PMID: 34313849

[14] Hyland ME Sodergren SC. Development of a new type of global quality of life scale, and comparison of performance and preference for 12 global scales. Qual Life Res. (1996) 5:469–80. doi: 10.1007/BF00540019, PMID: 8973126

[15] Schneider I Thumfart WF Pototschnig C Eckel HE. Treatment of dysfunction of the cricopharyngeal muscle with botulinum a toxin: introduction of a new, noninvasive method. Ann Otol Rhinol Laryngol. (1994) 103:31–5. doi: 10.1177/000348949410300105, PMID: 8291857

[16] Murry T Wasserman MS Carrau RL Castillo B. Injection of botulinum toxin a for the treatment of dysfunction of the upper esophageal sphincter. Am J Otolaryngol. (2005) 26:157–62. doi: 10.1016/j.amjoto.2004.11.010 15858769

[17] Kelly EA Koszewski IJ Jaradeh SS Merati AL Blumin JH Bock JM. Botulinum toxin injection for the treatment of upper esophageal sphincter dysfunction. Ann Otol Rhinol Laryngol. (2013) 122:100–8. doi: 10.1177/000348941312200205, PMID: 23534124 PMC3951150

[18] Alfonsi E Restivo DA Cosentino G De Icco R Bertino G Schindler A. Botulinum toxin is effective in the management of neurogenic dysphagia. Clinical-electrophysiological findings and tips on safety in different neurological disorders. Front Pharmacol. (2017) 8:80. doi: 10.3389/fphar.2017.00080 28275351 PMC5319993

